# Anti-Microbial Drug Metronidazole Promotes Fracture Healing: Enhancement in the Bone Regenerative Efficacy of the Drug by a Biodegradable Sustained-Release In Situ Gel Formulation

**DOI:** 10.3390/biomedicines12071603

**Published:** 2024-07-18

**Authors:** Shivali Duggal, Shivani Sharma, Nikhil Rai, Divya Chauhan, Vishal Upadhyay, Swati Srivastava, Konica Porwal, Chirag Kulkarni, Arun K. Trivedi, Jiaur R. Gayen, Prabhat R. Mishra, Naibedya Chattopadhyay, Subhashis Pal

**Affiliations:** 1 Division of Endocrinology, CSIR-Central Drug Research Institute, Council of Scientific and Industrial Research, Lucknow 226031, India; 2 Academy of Scientific and Innovative Research (AcSIR), Ghaziabad 201002, India; 3 Division of Pharmaceutics and Pharmacokinetics, CSIR-Central Drug Research Institute, Council of Scientific and Industrial Research, Lucknow 226031, India; 4 Division of Cancer Biology, CSIR-Central Drug Research Institute, Council of Scientific and Industrial Research, Lucknow 226031, India; 5 Division of Endocrinology, Metabolism and Lipids, Department of Medicine, Emory University, Atlanta, GA 30322, USA; 6 Division of Medical Research, SRM Medical College Hospital and Research Centre, SRM Institute of Science and Technology (SRM IST), Kattankulathur 603203, India

**Keywords:** osteogenic, anti-microbial drug, in situ gel formulation, osteoblast differentiation, fracture healing

## Abstract

Nitroimidazoles comprise a class of broad-spectrum anti-microbial drugs with efficacy against parasites, mycobacteria, and anaerobic Gram-positive and Gram-negative bacteria. Among these drugs, metronidazole (MTZ) is commonly used with other antibiotics to prevent infection in open fractures. However, the effect of MTZ on bone remains understudied. In this paper, we evaluated six nitroimidazole drugs for their impact on osteoblast differentiation and identified MTZ as having the highest osteogenic effect. MTZ enhanced bone regeneration at the femur osteotomy site in osteopenic ovariectomized (OVX) rats at the human equivalent dose. Moreover, in OVX rats, MTZ significantly improved bone mass and strength and improved microarchitecture compared to the vehicle-treated rats, which was likely achieved by an osteogenic mechanism attributed to the stimulation of the Wnt pathway in osteoblasts. To mitigate the reported neurological and genotoxic effects of MTZ, we designed an injectable sustained-release in situ gel formulation of the drug that improved fracture healing efficacy by 3.5-fold compared to oral administration. This enhanced potency was achieved through a significant increase in the circulating half-life and bioavailability of MTZ. We conclude that MTZ exhibits osteogenic effects, further accentuated by our sustained-release delivery system, which holds promise for enhancing bone regeneration in open fractures.

## 1. Introduction

Fractures necessitating surgical intervention pose multiple challenges to both patients and clinicians. Among these challenges, infection is the foremost condition, often leading to non-unions and the subsequent requirement for revision surgeries [1,2]. Non-unions, characterized by the failure of fractured bones to heal, prolong patient suffering and place a significant strain on them [3]. Although a combination of sterilization methods is used to minimize the risk of intra-operative contamination, prophylactic antibiotics have been the mainstay of preventing infection [4]. Depending on the requirements of the surgical procedures, antibiotics are used either systemically or locally in high amounts in the form of antibiotic-loaded cements or beads [5]. However, the impairment in bone regeneration could be deleterious to the correct healing of the fractures. For example, the quinolone group of antibiotics is one of the most widely used drugs for orthopedic applications but has adverse impacts on chondrocytes and osteoblasts, resulting in improper fracture healing in preclinical studies [6,7]. Tetracyclines promote bone mineralization; however, they inhibit osteoclast function and, at higher doses, induce osteoclast apoptosis [8]. Because osteoclasts are required to remodel the newly formed osteoid during fracture repair, inhibiting osteoclast function could impair the formation of the mature bone matrix [9,10].

In addition to the quinolone antibiotics and tetracyclines described above, gentamicin, an aminoglycoside, is widely used for treating open fractures. However, it has been reported to inhibit osteoblast function and decrease bone density in both preclinical and clinical settings [11,12]. Cephalosporins also impair fracture healing by inhibiting osteoblast function [13]. As such, bacterial infection inhibits bone regeneration, and an increase in bacterial burden inhibits bone matrix deposition [13,14]. Because of the vulnerability of osteoblasts to bacterial infections, the antibiotics used must be safe for the bone in general and osteoblasts in particular. Moreover, having an antibiotic with a bone-forming effect is an unmet clinical need. Anti-anaerobic antibiotics are crucial in preventing deep-seated infections during fracture healing by targeting anaerobic bacteria in bones, reducing risks like osteomyelitis and promoting successful healing.

Metronidazole (MTZ) belongs to the nitroimidazole class and is a first-line anti-anaerobic drug. It is also used for amebiasis and surgical prophylaxis [15]. Moreover, MTZ is clinically used for the management of osteomyelitis along with other groups of antibiotics, such as quinolones, cephalosporins, and aminoglycosides [16]. The maximum human dose is 4 g for 7–10 days; however, for osteomyelitis, MTZ is recommend up to 1500 mg daily by the intravenous route [17,18]. MTZ is used prophylactically in combination with other antibiotics in open fractures to prevent of infection [19]. However, its effect on bone cells and the skeleton is not known.

In this study, our initial step involved screening a library of approved nitroimidazole drugs for clinical use to identify those exhibiting osteogenic effects in vitro. Out of six drugs, MTZ showed the least osteogenic EC_50_; so, we assessed its skeletal effects in rats, first on bone regeneration at the femur osteotomy site and then in osteopenic rats. The skeletal effect was evaluated by studying bone mass, microarchitecture, bone formation, bone turnover, and bone strength. Subsequently, we evaluated whether lowering the dosage to levels below the maximum human dose could still promote effective bone healing, aiming to minimize drug burden. To achieve this goal, we developed a long-acting drug delivery system designed to modulate the release rate of MTZ and assessed its fracture healing efficacy.

## 2. Materials and Methods

### 2.1. Reagents and Chemicals

MTZ (purity > 99.0%) was purchased from TCI (India) Pvt. Ltd. (Chennai, India). Collagenase, cell culture medium, and all other fine chemicals were acquired from Sigma Aldrich (St. Louis, MO, USA). FBS and cell culture supplements were purchased from Invitrogen (Waltham, MA, USA). SYBR green and cDNA synthesis kit were purchased from Thermofisher (Waltham, MA, USA). The remaining analytical-grade HPLC or liquid chromatography–mass spectrometry (LC-MS) solvents were purchased from Merck (Mumbai, India).

### 2.2. Animal Experiments

The Institutional Animal Ethics Committee (permission number CDRI/IAEC/2016/72) granted prior approval for all animal experimentation, which were carried out in compliance with the guidelines. The study’s animals were acquired from NLAC, CDRI, and they underwent a 12 h cycle of darkness and light with regulated humidity levels (50–60%) and temperature (23–25 °C).

### 2.3. Femur Osteotomy

Femur osteotomy was carried out in adult female Sprague Dawley (SD) rats (200 ± 20 g, 3 months old) and 3-month post-ovariectomized osteopenic SD rats (280 ± 20 g, 6 months old) by inserting a drill bit with a diameter of 0.8 mm from the anterior to the posterior mid-diaphysis of the right femur.

The human equivalent dose for rats is 235 mg/kg; half of this amount, 117 mg/kg, was used for the study [19]. Doses of MTZ (117 mg/kg and 235 mg/kg) were administered daily by the oral route for 12 days. For the formulation study, MTZ@In situ gel and unformulated MTZ were administered every 3 days (total of 4 injections) for a period of 12 days via the subcutaneous route in the osteotomy animal. Each rat received 20 mg/kg, s.c. of bone-seeking fluorochrome calcein 24 h before sacrifice in order to evaluate the new bone formation at the fractured callus [20].

### 2.4. Ovariectomy

The 10-month-old (265 ± 20 g) SD rats were ovariectomized (OVX) bilaterally and left for 3 months to develop osteopenia. Ovary-intact sham-operated animals were used as a control. A live animal micro-CT was performed to confirm osteopenia, followed by dividing the animals into 3 groups: Sham + vehicle (water, oral), OVX + vehicle (water, oral), and OVX + MTZ (235 mg/kg, oral). Each group had 9 rats, and treatment were administered for 3 months. Calcein (20 mg/kg, s.c.) was injected twice before sacrificing at 10-day intervals [21].

### 2.5. Culture of Rat Calvarial Osteoblasts (RCOs)

Sequential digestion was performed to isolate calvarial osteoblast cells from neonatal rat pups. Briefly, calvariae were harvested from ten to twelve 1–2-day old rat pups, cleaned, and subjected to five sequential enzymatic digestions (0.1% dispase and 0.1% collagenase I) of 10–15 min each, followed by culturing of the harvested cells in α-MEM supplemented with 10% FBS [22].

### 2.6. ALP Assay

RCOs were seeded in 24-well plates (1 × 10^4^ cells/well), and the cells were treated for 48 h in osteoblast differentiation media (α-MEM supplemented with 10 mM β-glycerophosphate and 50 μg/mL ascorbic acid) with the imidazole group of chemicals (10^−11^ M–10^−6^ M).

A total of 2 mg/mL of para-Nitrophenyl phosphate in diethanolamine buffer was used to measure the ALP activity at a 405 nm wavelength [23].

To evaluate the osteogenic activity of MTZ, rat and mouse bone marrow stromal cells (BMSCs) were seeded in 24-well plates (5 × 10^4^ cells/well) and treated with either MTZ (1 nM) or the positive control BMP2 (100 ng/mL) for 48 h in a differentiation medium, followed by measuring the ALP activity at 405 nm [24].

### 2.7. BrdU Assay

Rat and mouse bone marrow stromal cells (BMSCs) were seeded at a density of 4000 cells/well in a 96-well plate for cell proliferation and treated with 1 nM of MTZ for 48 h in a growth medium (α-MEM containing 10% FBS). Following the MTZ treatment, the cells were incubated for 4 h with bromodeoxyuridine (BrdU) before termination. Cell proliferation was assessed at 450 nm using the Roche Diagnostics BrdU ELISA kit, in accordance with the manufacturer’s protocol, using a reference wavelength of 690 nm.

### 2.8. Nodule Mineralization and Alizarin Staining

Rat and mouse BMSCs were seeded at a density of 1 × 10^6^ cells/well in a 6-well plate, vehicle or MTZ (1 nM) treatment was conducted, and the media were changed every alternate day for 21 days using a differentiation medium [10% FBS, β-glycerophosphate (10 mM), ascorbic acid (50 µg/mL), and dexamethasone (100 nM) in α-MEM].

In a different set of experiments, 3 × 10^6^ C3H10T1/2 cells were seeded in a 60 mm culture dish. Then, 24 h post-seeding, the vehicle or MTZ treatment was conducted, and the media were changed every alternate day for 21 days using a differentiation medium.

Cells were fixed with 10% formalin for 15 min and stained with Alizarin Red for 1 h after 21 days of culture. Moreover, 10% cetylpyridinium chloride (Sigma #C0732) was used to quantify mineralized nodules at a 595 nm wavelength.

### 2.9. Quantitative Real-Time Polymerase Chain Reaction

Quantitative real-time polymerase chain reaction (qPCR) was performed to assess the expression of osteogenic genes in rat and mouse BMSCs and osteopenic animals’ femurs. Rat and mouse BMSCs were seeded at a density of 1 × 10^6^ cells/well in a 6-well plate, and the vehicle or MTZ (1 nM) treatment was conducted for 48 h. For the osteopenic animals’ femur analysis, post-treatment femur bones were collected, bone marrow was flushed out, and the bone sample was crushed in liquid nitrogen. RNA was isolated using Trizol reagent, and cDNA was synthesized from 2 μg total RNA by using a RevertAid cDNA synthesis kit (Fermentas, Austin, TX, USA) [25]. SYBR green chemistry was used to perform quantitative determinations of the relative gene expression. GAPDH was used as the internal control. Primers were designed using the Universal ProbeLibrary (Roche Applied Science, Penzberg, Germany) for the following genes: Runx2, BMP2, and TRAP. Primer sequences are listed in Table 1. All genes were analyzed using the Light Cycler 480 (Roche Molecular Biochemicals, Indianapolis, IN, USA).

### 2.10. Wnt/β-Catenin Reporter Assay

The effect of MTZ on β-catenin signaling was assayed using a TOP-Flash reporter plasmid that contained a minimal TA viral promoter coupled to β-catenin-responsive TCF/LEF-binding sites upstream of a firefly luciferase gene (a kind gift from Prof. Yosef Yarden, WIS, Israel). Briefly, HEK293T cells were transfected with 0.5 µg of the TOP-Flash plasmid. Additionally, the GFP-encoding plasmid, pEGFP, was transfected as a control for transfection efficiency. The transfected cells were treated with rhWnt3a (300 ng/mL), sclerostin peptide (6 µg/mL), and MTZ for 24 h, followed by cell lysis (1 M tris-HCl pH 8 and 0.1% Triton X-100) for 30 min at RT. In white opaque plates, the lysate and 100 µL of luciferase assay buffer (10 mM KH_2_PO_4_, 2 mM ATP, 1 mM D-luciferin, NaCl, and MgCl_2_) were added, and luminescence was measured. Luminescence values were normalized by EGFP plasmid fluorescence value, and the fold-change was calculated relative to the control.

### 2.11. Immunoflorescence

RCOs were grown on Lab-Tek Chamber Slides (Nunc, Penfield, NY, USA) to 40–50% confluence for 24 h. After treatment with MTZ at different time points, cells were fixed with 4% formaldehyde, followed by permeabilization with 0.1% Triton™ X-100 for 15 min, blocked with 2% goat serum for 2 h, and labeled with primary antibody against β-catenin (Cell Signaling Technology, Danvers, MA, USA, catalog number 9562S) (1:100) overnight at 4 °C in a moist chamber. Alexa Fluor® 594 (Abcam, Cambridge, UK, catalog number ab150080) was used as a secondary antibody (1:200 dilution) for 2 h at room temperature [26]. The images were captured at 20× using a fluorescence microscope (Nikon Eclipse Ni H600L microscope, Tokyo, Japan).

### 2.12. Western Blotting

Western blotting was performed as previously described [27]. Briefly, whole-cell extracts (WCEs) were prepared at indicated time points using RIPA lysis buffer, and aliquots of 30 μg protein per lane were loaded and resolved on SDS-PAGE (10%), followed by blotting onto the PVDF membrane (Millipore Inc., Burlington, MA, USA). The membrane was blocked in 1% BSA, followed by immunoblotting with primary antibodies: Runx2 (ab 76956) (1:1000), pGSK (9323S) (1:1000), and GSK (9315S) (1:1000) at 4 °C overnight. Anti-rabbit HRP secondary antibody (Sigma, catalog number A0545) (1:3000) was used for Runx2, pGSK3β, and GSK3β. Bands were detected by incubating the membranes with ECL substrate (Thermo Fisher Scientific, Waltham, MA, USA) and developed on ChemiDoc (Image Quant LAS 4000, GE Healthcare, Hatfield, UK). Blots were re-probed after washing with mild stripping buffer (1.5% Glycine, 0.1% SDS, and 1% Tween 20).

### 2.13. Formulation of PLGA-Based Biodegradable In Situ Gel of MTZ

The polymer solution (PLGA; 50:50) was dissolved in 60% NMP (*w*/*w*) to make the final PLGA concentration (30% *w*/*w*). The solution was left for stirring at 3000 rpm on a stirring hotplate at 60 °C. After this, MTZ was added into the clear polymeric solution at a 10% weight ratio. After thorough stirring, the resultant fo rmulation was brought to room temperature and stored for further analysis, including size, Zeta potential, polydispersity index (PDI), and drug content. The Zeta potential was measured by the Malvern Zetasizer Software version 7.13 in conjunction with the Nano-Zs 2000 equipment from Malvern Instruments, Malvern, UK. Dynamic light scattering (DLS) was used to analyze the formulation’s particle size, and distribution/polydispersity index (PDI) at room temperature. PDI is a unitless value used to describe the size distribution of particles. PDI < 0.4 typically indicates a narrow size distribution, meaning most particles have a similar size. This is ideal for injectable applications where a consistent particle size is crucial. The in-situ gel-based formulation remained liquid at room temperature, and after preparation, it was filtered through a 0.22-micron membrane filter to ensure its sterility.

### 2.14. Determination of Drug Content

The drug content of the final formulation was assessed using a modified protocol: a known amount of the formulation was vortexed in methanol, centrifuged (4000 rpm, 25 °C for 15 min), and filtered through a 0.2 μm syringe filter [28]. The drug in the supernatant was analyzed spectrophotometrically (294 nm) using the following formula: Percentage of Drug = (Actual weight of drug)/(Theoretical weight of drug) × 100.

### 2.15. Morphological Evaluation

For a detailed morphology evaluation, the lyophilized sample was mounted on an aluminum stub. A thin coating of gold was applied using a sputter coater to enhance the electrical conductivity. The sample was then examined under SEM (FEI Quanta 250 SEM) at 10 kV with a secondary electron (SE) detector, capturing topographical images at various magnifications.

### 2.16. Fourier Transform Infrared Spectroscopy (FTIR)

Fourier transform infrared spectrophotometer (FTIR) (PerkinElmer-Spectrum Two FTIR Spectrometer, Waltham, MA, USA) was used to record the spectra of the samples (MTZ, PLGA, NMP, and MTZ@In situ gel), and the analysis was performed using the PerkinElmer Spectrum II software OPPUS 8.8.4. The samples were scanned over the range from 4000 to 400 cm^−1^.

### 2.17. Evaluation of MTZ Release and Its Kinetics

To evaluate the release kinetics profile of MTZ, a formulation containing 10 mg equivalent of MTZ was injected into 200 mL of the PBS medium (37 ± 0.5 °C) using a 20-gauge needle. The formulation immediately turned into a gel upon contact with the dissolution medium. The dissolution vials were placed on a reciprocating bath-shaker and left for continuous shaking throughout the dissolution study. A total of 2 mL of aliquots were withdrawn and replaced with an equal volume of fresh PBS to maintain the sink condition. The dissolution study was conducted for a duration of 24 h. The samples were filtered through a syringe filter (0.22 μm). The MTZ concentrations were analyzed spectrophotometrically (294 nm) after appropriate dilution, with PBS as a blank for background correction. The measured concentrations were presented as the mean ± standard deviation (SD).

To understand the drug release kinetics, data obtained from the release study were fitted into various kinetics models: zero-order, first-order, Higuchi, Korsmeyer–Peppas, and Hixson–Crowell. The DD solver Excel add-in program was utilized to compare and identify the mechanism of drug release.

### 2.18. Bioanalytical Method Development Using LC-MS/MS

Mass spectrometric detections were carried out using an AB Sciex 4000 QTRAP LC-MS/MS, Redwood City, CA, USA) instrument fitted with an electrospray ionization source. Separation was conducted through X Bridge^TM^ Shield RP 18 (Waters, MA, USA) (5 µm, 4.6 × 50 mm) column. The mobile phase consisted of acetonitrile: 0.1% formic acid in the ratio of 90:10 (*v*/*v*) at a flow rate of 0.5 mL/min. The ion spray voltage was set at 4500 V. The source parameters ion source gas (GS1), ion source gas (GS2), and curtain gas were optimized to 10, 20, and 10 psi, respectively, while the collision gas (CAD) was set to medium. The compound parameters, such as declustering potential (DP), collision energy (CE), entrance potential (EP), and collision exit potential (CXP), were set at 55, 21, 10, and 6 V for the internal standard carbamazepine (CBZ) and 96, 30, 10, and 10 V for MTZ, respectively. The MS was operated in the ESI positive-ion mode, and the detection of the ions was performed in the multiple reaction monitoring (MRM) mode, monitoring the transition of *m*/*z* 172.06 precursor ion [M + H]+ to the *m*/*z* 128.08 product ion for IS and *m*/*z* 236.95 precursor ion [M + H]+ to the *m*/*z* 194.04 product ion for compound, respectively.

### 2.19. Pharmacokinetic (PK) Studies

After overnight fasting, the animals were divided into two groups (n = 4) based on the treatment: MTZ and the MTZ@In situ gel (dose = 40 mg/rat equivalent to 160 mg/kg, for 250 ± 10 g rat, administered via the subcutaneous route). An MTZ suspension was prepared in water. Blood samples were collected at predetermined time points (0.25 to 48 h). Plasma was isolated by centrifugation at 10,000 rpm for 10 min using a Sigma 1–15K centrifuge (Frankfurt, Germany) and stored at −70 ± 10 °C until bioanalysis.

To extract MTZ from plasma samples, the protein precipitation method was used. In this method, plasma samples (50 μL) were taken (50 ng/mL of carbamazepine (CBZ) as the internal standard) and vortexed at 2000 rpm for 10 min. Subsequently, the samples were centrifuged at 10,000 rpm for 10 min. The supernatant (100 µL) was taken for LC-MS/MS analysis. Data acquisition and quantitation were performed using analyst software. The PK parameters were determined using the Phoenix™ 6.3 WinNonlin (Pharsight Corporation, Mountain View, CA, USA) software as a noncompartmental model approach.

### 2.20. Micro-CT Analysis

A SkyScan 1076 μCT scanner (SkyScan, Ltd., Kartuizersweg, Kontich, Belgium) was used to perform a high-resolution μCT for the two-dimensional and three-dimensional assessment of excised bones. The rat bone scanning process used an isotropic voxel pixel size of 18 μm and an X-ray source of 70 kV and 140 mA. Utilizing the SkyScan Nrecon software (Version: 1.7.0.4), cross-sectional reconstruction was performed. In order to examine the trabecular region of the femur and tibia, 100 slices were used to draw the region of interest (ROI), which was limited to the secondary spongiosa region 1.5 mm from the growth plate’s distal border. The primary spongiosa and cortical bone were not included in the ROI. A ROI was drawn on 70 slices of the mid-vertebral region for the L5 vertebral study. The Batman software (Version: 1.16.8.0+), with trabecular (3D) bone analysis programs was used for quantification. Two distinct density phantom rods were utilized to calibrate for the BMD calculation: the high-density phantom rod had a BMD value of 0.75 g-HA/cm^3^, while the low-density phantom rod had a BMD value of 0.25 g-HA/cm^3^ [25].

### 2.21. Bone Strength Test

The mechanical compression strength of the femur head was examined by a compression test with a bone strength tester, TK 252C (Muromachi Kikai Co., Ltd., Tokyo, Japan), as described before [29].

### 2.22. Bone Histomorphometry

Two calcein injections were spaced ten days apart for double calcein labeling to perform dynamic histomorphometric assessments. A previously published protocol was used to calculate the bone formation rate/total bone surface (BFR/BS), mineralizing surface/bone surface (MS/BS), and mineral apposition rate (MAR) [30]. Briefly, we measured periosteal perimeters, single-labeled surface (sLS), double-labeled surface (dLS), and interlabeled thickness (IrLTh), and these data were used to calculate MS/BS, MAR, and BFR, respectively, as follows: MS/BS = (1/2sLS + dLS)/BS; MAR = IrLTh/10 days (μm/day); and BFR/BS = MAR × MS/BS (μm^3^/μm^2^/day).

### 2.23. Bone Turnover Markers

The levels of serum procollagen type I N-terminal peptide (P1NP) and serum cross-linked C-telopeptide of type I collagen (CTX-I) were assessed using ELISA (MyBioSource, San Diego, CA, USA), in accordance with the manufacturer’s protocol.

### 2.24. Statistical Analysis

The mean ± standard error of the mean (SEM) was used to express the data. Using GraphPad Prism 5, a post hoc Dunnett’s multiple comparison test of significance was performed to examine statistical differences between the various treatment groups. A *t*-test was used to analyze data within two groups and determine the statistical significance.

## 3. Results

### 3.1. Effect of Nitroimidazole Drugs on ALP Activity

**Figure 1:**
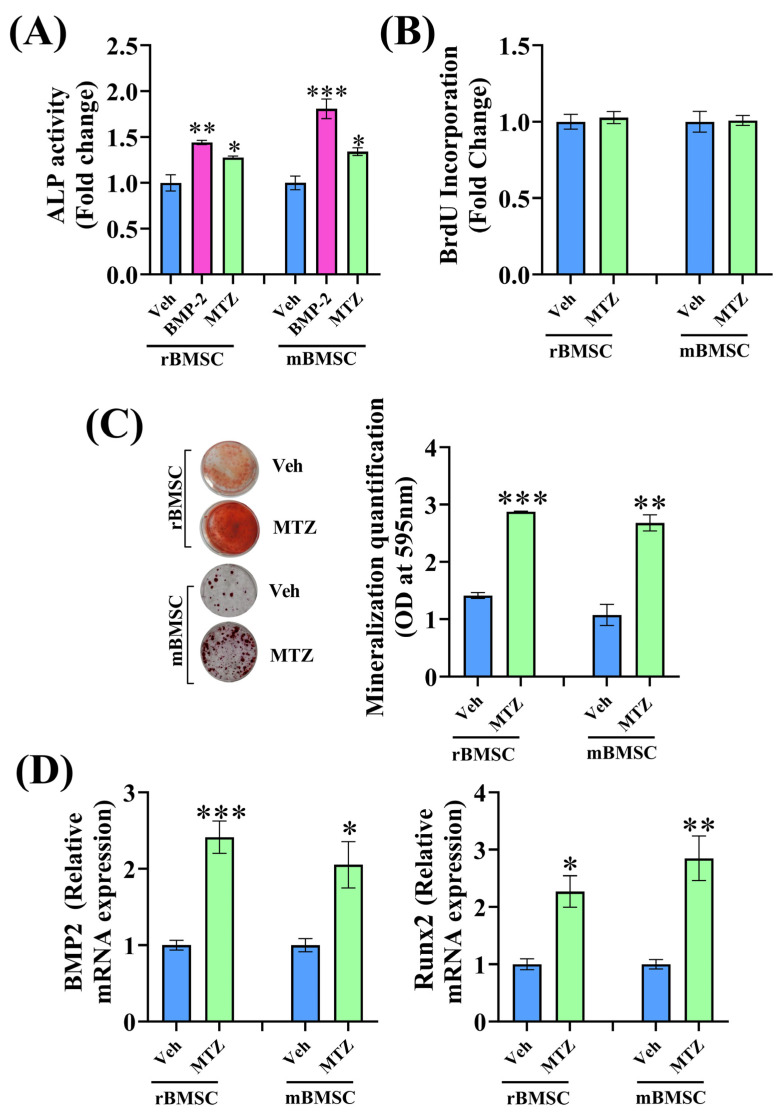
Metronidazole (MTZ) has osteogenic activity. (**A**) rBMSCs and mBMSCs were treated with 1 nM of MTZ in the differentiation medium for 7 days, and ALP activity was measured. BMP-2 (100 ng/mL) was used as a positive control. (**B**) rBMSCs and mBMSCs were exposed to the vehicle (0.1% DMSO) or MTZ (1 nM) for 24 h, and the proliferation rate was measured by BrdU incorporation by ELISA. (**C**) Exposing rBMSCs and mBMSCs to MTZ for 21 days in the differentiation medium significantly increased mineralized nodules compared to the vehicle, as assessed by Alizarin Red S staining. Left panel shows representative Alizarin S dye-stained nodules and right panel shows the quantified data after dye extraction by cetylpyridinium chloride (CPC). (**D**) rBMSCs and mBMSCs treated with 1 nM of MTZ for 24 h resulted in an increase in the mRNA levels of BMP-2 and Runx-2. All values are expressed as mean ± SEM. * *p* < 0.05, ** *p* < 0.01, and *** *p* < 0.001 compared to the vehicle (0.1% DMSO).

RCOs were treated with clinically used nitroimidazole drugs for 48 h to assess osteoblast differentiation by ALP activity, and BMP2 was used as a positive control. Secnidazole (anti-infective), tinidazole (anti-trichomoniasis), ornidazole (anti-protozoan), and ronidazole (veterinary anti-histomoniasis) had no effect on ALP activity. However, both etanidazole (radiosensitizer) and MTZ (an antibiotic used in type III open fractures) significantly increased ALP activity compared to the control (vehicle) (Table 2). Comparing etanidazole and MTZ, the latter exhibited an EC_50_ nearly 330-fold lower, leading us to select this drug to further investigate its impact on bone. In both rat BMSCs (rBMSCs) and mouse BMSCs (mBMSCs), MTZ (1 nM) increased ALP activity seven days post-treatment compared to the vehicle group (Figure 1A). MTZ did not affect the proliferation of rBMSCs and mBMSCs at 1 nM (Figure 1B).

### 3.2. MTZ Increases Osteoblast Differentiation

**Figure 2:**
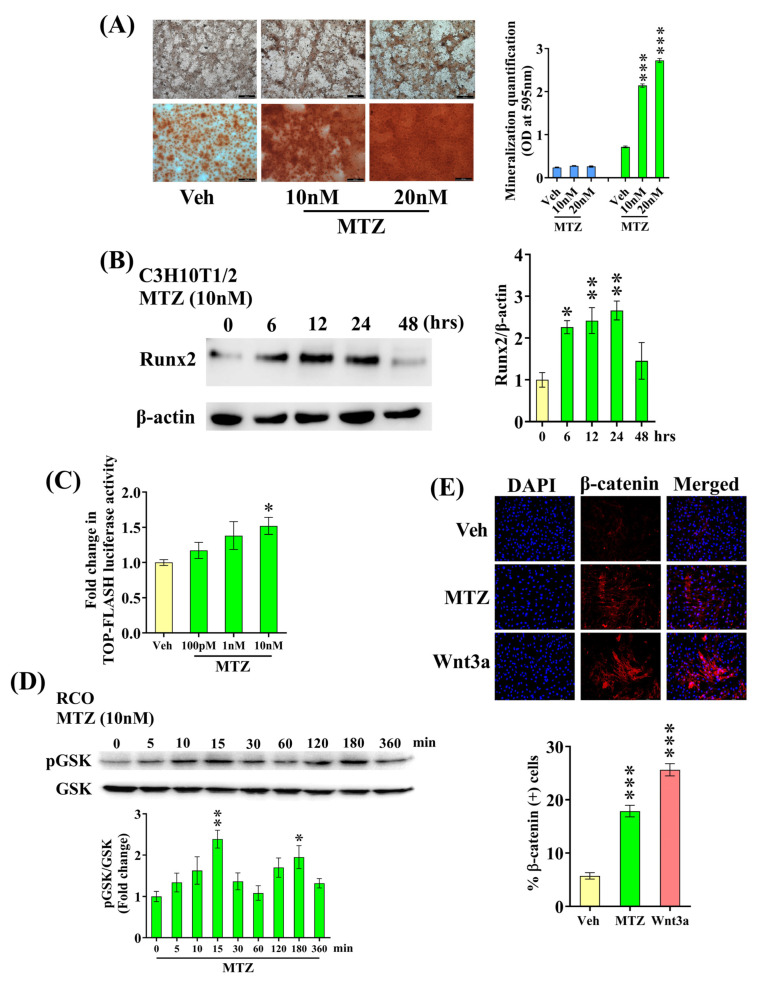
MTZ stimulates the Wnt signaling pathway in osteoblasts. (**A**) Exposing C3H10T1/2 cells to MTZ for 21 days in the differentiation medium significantly increased mineralized nodules compared to the vehicle, as assessed by Alizarin Red S staining. Left panel shows representative Alizarin S dye-stained nodules and right panel shows the quantified data after dye extraction by CPC. (**B**) Exposing C3H10T1/2 cells to MTZ (10 nM) increased Runx2 expression. Left panel shows the representative blots and right panel showing the quantification of Runx2 expression from 3 independent experiments. (**C**) TOP-Flash reporter assay to evaluate the Wnt transactivation by MTZ. (**D**) Exposing RCO to MTZ (10 nM) showed an early increase in p-GSK levels. Top panel shows the representative blots and bottom panel shows the quantification of p-GSK and GSK expression from 3 independent experiments. (**E**) Exposing RCO to MTZ (10 nM) increased β-catenin nuclear translocation. Wnt3a was used as a positive control. Top panel shows the representative images and bottom panel shows the quantification of the data. All values are expressed as the mean ± SEM (n = 3). * *p* < 0.05, ** *p* < 0.01, and *** *p* < 0.001 compared to the vehicle (water) or as indicated.

In vitro, osteogenic differentiation is most effectively assessed by observing the ability of osteoblasts to generate mineralized nodules when cultured in media supplemented with β-glycerophosphate and ascorbate (differentiation medium). MTZ stimulated osteogenic differentiation and mineralized nodule formation by rBMSCs and mBMSCs 21 days post-treatment (Figure 1C). Also, MTZ increased the mRNA levels of osteogenic genes, including BMP-2 and Runx2, in BMSCs (Figure 1D). To further validate our data, we measured MTZ-mediated mineralization in C3H10T1/2 cells (a murine MSC cell line) and observed that 21 days of MTZ treatment promoted mineralization in the presence of a differentiation medium (Figure 2A). We also found that Runx2 expression was increased in C3H10T1/2 cells by the MTZ treatment (Figure 2B).

### 3.3. MTZ Stimulates Wnt Pathway in Osteoblasts

Given the osteogenic effect associated with the activation of canonical Wnt signaling, we investigated whether MTZ induces this pathway using a TOP-Flash reporter assay. This assay measures Wnt pathway activity by detecting luciferase expression driven by TCF/LEF-binding sites within the reporter construct. We observed that MTZ increased TOP-Flash reporter activity (Figure 2C). We also showed that MTZ increased GSK3β phosphorylation in a time-dependent manner, showing a maximum effect at 15 min (Figure 2D). Moreover, MTZ induced β-catenin nuclear translocation in osteoblasts, mirroring the effect of Wnt3a (Figure 2E), a hallmark downstream event of Wnt pathway activation. Together, these data establish that MTZ stimulates the Wnt pathway in osteoblasts.

### 3.4. MTZ Enhances Bone Regeneration at the Osteotomy Site

**Figure 3:**
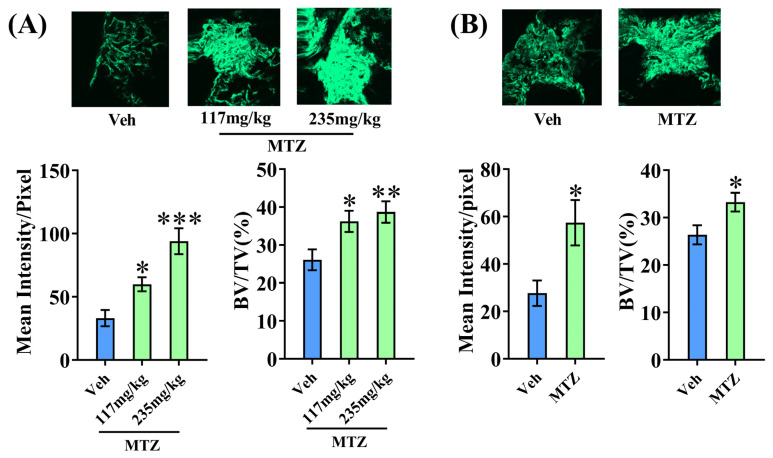
MTZ treatment increased new bone formation at the fracture site. (**A**) Top panel showing representative confocal images (10×) of calcein labeling at the callus site after 12 days of oral dosing of MTZ. Bottom panel showing quantification of the mean intensity of calcein label and bone volume (BV/TV) quantification. (**B**) Top panel showing representative confocal images (10×) of calcein labeling at the callus site after 12 days of oral dosing with 235 mg/kg MTZ in OVX rats. Bottom panel showing the quantification of the mean intensity of calcein label and bone volume (BV/TV) quantification. All values are expressed as mean ± SEM (n = 6 rats/group). * *p* < 0.05, ** *p* < 0.01, and *** *p* < 0.001 compared to the vehicle (water).

The ability of a drug to enhance bone regeneration at the osteotomy site is a surrogate of its in vivo osteogenic impact, which was investigated for MTZ using a rat femur osteotomy model. The human equivalent dose for rats is 235 mg/kg; half of this amount, 117 mg/kg, was used for the study [19]. MTZ at both doses increased calcein intensity and bone volume at the osteotomy site; however, the maximum effect was shown with the 235 mg/kg dose (Figure 3A), which was used for the subsequent studies.

Because bone regeneration is impaired in osteopenic conditions such as OVX, we used this model to study the osteogenic ability of MTZ. Osteopenia was confirmed in OVX rats when starting the MTZ treatment (235 mg/kg), and data showed that MTZ increased calcein intensity and bone volume at the callus site compared to the control group (Figure 3B).

### 3.5. MTZ Restores OVX-Induced Bone Loss and Strength

**Figure 4:**
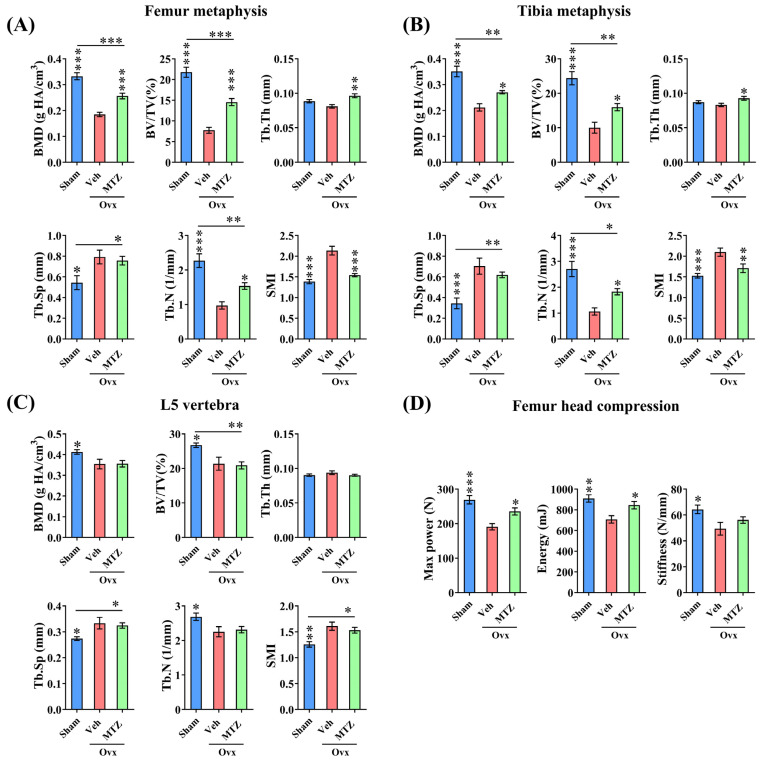
MTZ treatment (235 mg/kg) improved trabecular microarchitecture in OVX osteopenic rats. (**A**) MTZ treatment for 3 months increased bone mineral density (BMD), bone volume (BV/TV), trabecular thickness (Tb.Th), and trabecular number (Tb.N) and decreased structure model index (SMI) in femur metaphysis compared to the vehicle (water)-treated animals. (**B**) MTZ treatment for 3 months improved the tibia metaphysis’s trabecular microarchitecture compared to the vehicle (water)-treated rats. (**C**) MTZ treatment for 3 months failed to improve L5 trabecular microarchitecture compared to the vehicle-treated rats. (**D**) Femur head compression showed increase in maximum load-bearing capacity and energy to failure after 3 months of MTZ treatment compared to the vehicle-treated osteopenic rats. All values are expressed as mean ± SEM (n = 8 rats/group). * *p* < 0.05, ** *p* < 0.01, and *** *p* < 0.001 compared to the vehicle (water) or as indicated.

The bone regenerative effects of MTZ observed at the osteotomy site prompted us to explore whether the drug exhibits bone anabolic effects in the OVX rats. In proximal femur and tibia metaphyses, BMD and bone volume (BV/TV) were reduced in OVX, and the MTZ treatment significantly increased these two parameters in the OVX group (Figure 4A,B). MTZ restored trabecular spacing (Tb.sp) to sham levels at both sites, attributable to the complete restoration of trabecular thickness (Tb.Th) to the sham group and a significant enhancement in the trabecular number (Tb.N) in the OVX group (Figure 4A,B). The Structure Model Index (SMI) reflects the trabecular bone’s geometry, balancing plate-like (resisting compression) and rod-like (resisting bending) structures. Since trabecular bones experience mainly compressive loads, a higher proportion of rod-like structures increases SMI values. In our study, SMI values were higher in OVX rats than in the sham group, but MTZ reduced them to sham levels (Figure 4A,B). However, we observed that, at vertebra (L5), MTZ failed to restore the trabecular bone parameters in the osteopenic animals (Figure 4C). These data suggest a site-specific bone restoration effect of MTZ in osteopenic rats.

In line with the enhanced bone mass and improved trabecular microarchitecture, our findings indicate an increased capacity to withstand compression failure in the femur head of OVX rats treated with MTZ. Maximum power indicates resistance to deformation or failure, and energy indicates that the bone’s resilience was decreased in the OVX group and MTZ reversed both parameters to sham levels. Stiffness indicates the bone’s rigidity, which was decreased in the OVX group; however, MTZ failed to increase it (Figure 4D).

### 3.6. In Vivo Osteogenic Effect of MTZ

**Figure 5:**
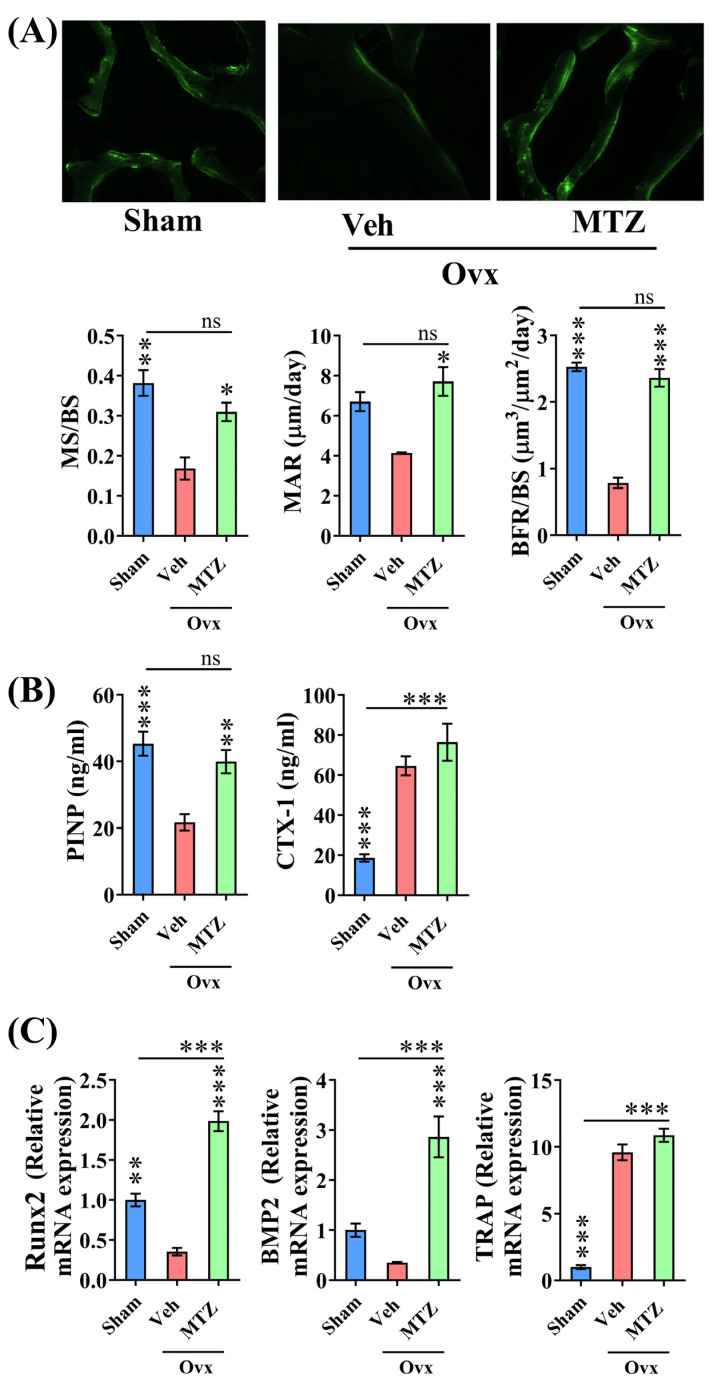
MTZ treatment (235 mg/kg) improved trabecular microarchitecture in OVX osteopenic rats via an osteoanabolic mechanism. (**A**) Upper panel shows a representative image of double calcein labeling. Lower panel indicating quantified mineralized bone surface (MS/BS), mineral apposition rate (MAR), and bone formation rate (BFR). (**B**) Serum bone turnover marker analysis demonstrates a significant increase in serum PINP after the MTZ treatment without altering serum CTX-1 compared to the vehicle-treated rats. (**C**) Bone mRNA data analysis reveals a significant increase in Runx2 and BMP2 expression in MTZ-treated bones, without altering TRAP expression compared to the vehicle-treated rats. All values are expressed as mean ± SEM (n = 3–6 rats/group). ns, *p* > 0.05, * *p* < 0.05, ** *p* < 0.01, and *** *p* < 0.001 compared to the vehicle (water) or as indicated.

Assessing surface-referent bone formation through time-spaced double labeling with a bone-seeking dye (calcein) is widely regarded as the gold standard for evaluating the osteoanabolic effects of a drug. All three parameters of bone formation, including MS/BS, MAR, and BFR, were decreased in the femur metaphysis of OVX rats, and MTZ reversed all parameters to sham levels (Figure 5A).

In addition to the rise in surface-referent bone formation, MTZ restored serum bone formation marker P1NP levels, which were significantly reduced in the OVX group compared to the sham group, to levels comparable to those of the sham group (Figure 5B). Our result show that the osteogenic genes, including Runx2 and BMP-2 levels were decreased in the bones of OVX rats, but their levels were comparable between the sham and MTZ groups (Figure 5C).

The serum resorption marker CTX-1 was increased in OVX rats; however, MTZ failed to reduce it (Figure 5B). Moreover, TRAP mRNA levels in bones that were elevated in OVX rats were comparable to those of the OVX + MTZ group (Figure 5C).

### 3.7. Development of Biodegradable-Based In Situ Gel (MTZ@In Situ Gel)

**Figure 6:**
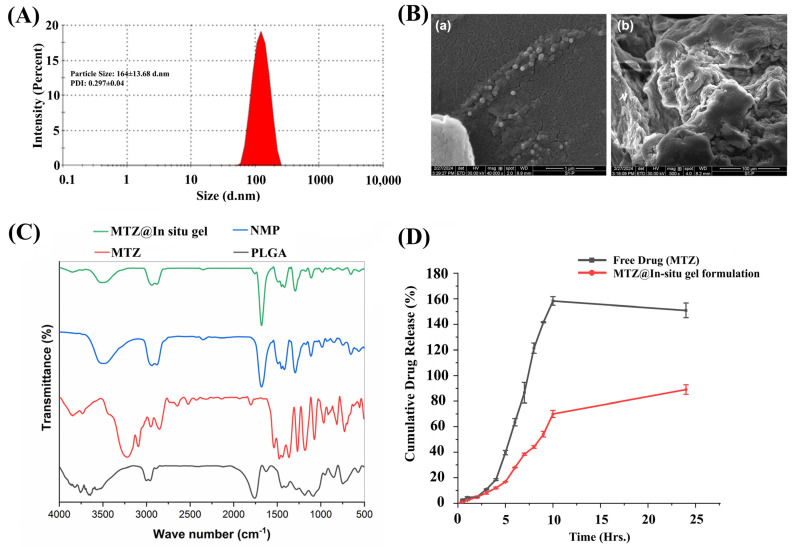
Characterization of the biodegradable PLGA-based in situ gel formulation of 40 mg MTZ. (**A**) Particle size distribution of in situ gel nanoparticle of MTZ. The average globular size and PDI of MTZ@In situ gel were found to be 164 d.nm and 0.297, respectively. (**B**) Scanning electron microscope (SEM) image of the PLGA-based in situ gel formulation of MTZ. (**a**). Liquid state. (**b**). Dried state. (**C**) FTIR spectra of MTZ, PLGA, NMP, and MTZ@In situ gel formulation. (**D**) In vitro cumulative drug release vs. time plot for free drug (MTZ) and MTZ@In situ gel formulation in PBS. Data are represented as the mean ± SD.

The administration of MTZ to rats at 235 mg/kg/day for bone regeneration (as shown in Figure 3) resulted in an absolute amount of 47 mg/rat/day, based on an average weight of 200 g. For the formulation study to augment the efficacy of MTZ, we approximated the daily dosage to 40 mg/rat. We developed a novel N-methyl pyrrolidone (NMP)-based thermo-responsive in situ gel for MTZ (MTZ@In situ gel) depot formulation. This formulation undergoes a phase transition from a solution to a gel state upon administration, triggered by temperature. The prototype formulation upon injection becomes solidified as an implant at the site of administration to release the drug in a sustained manner. The system was found to be stable with a high payload of MTZ (40 mg) along with controlled particle size. The particle size and distribution index were 164 ± 13.68 and 0.29 ± 0.04, respectively (Figure 6A). The drug content was quantified as 90 ± 0.254%.

### 3.8. Morphological and Functional Characterization by Scanning Electron Microscopy (SEM) Fourier Transform Infrared Spectroscopy (FTIR)

Figure 6B (a,b) depicts the morphological changes in nanogel particles before and after gelation. The striking difference was observed after gelation, where the spherical nanogel particles form a cross-linked network with an irregular internal structure. The observed changes might be attributed to the rapid displacement of water-miscible NMP with surrounding water. This sudden exchange of water causes the formation of a cross-linked network structure.

FTIR was employed to investigate the possible interactions of MTZ with PLGA and NMP solvent by recording the shifting or disappearance of absorption bands of the samples (Figure 6C). The pure MTZ showed characteristic bands at 3220 cm^−1^ (-C-H stretching), 2600–3100 cm^−1^ (-C-H stretching), 1540 cm^−1^ (-C=N stretching), 1400–1500 cm^−1^ (-N=O asymmetric stretching), 1363 cm^−1^ (-CH3 bending), 1100–1270 cm^−1^ (-C-O stretching), 1070 cm^−1^ (-C-N stretching), and 732 cm^−1^ (=C-H stretching). PLGA showed absorption bands at 3000–3100 cm^−1^ (-OH group), 2800–3000 cm^−1^ (-C-H stretching), strong peek at 1763 cm^−1^ (C=O stretching), 1000–1200 cm^−1^ (-C-O stretching), and 853 cm^−1^ (-C-H bending). Any residual NMP (e.g., C=O stretching at 1681 cm^−1^) were also monitored. No significant change was observed in the case of MTZ@In situ gel FTIR spectra. Peaks in the range of 2800–3000 cm^−1^ are typically assigned to NMP solvent peaks. Similarly, peaks in the 1200–1500 cm^−1^ range arise from the functional groups present in the MTZ molecule. Upon evaluating the FTIR spectrum of the MTZ@In situ gel formulation, we observed a slight shift of PLGA absorption bands at 1763 cm^−1^. This might be attributed to the formation of intermolecular hydrogen bonds between the nitro (NO_2_) group of metronidazole and the carbonyl group (-C=O) of PLGA.

### 3.9. Evaluation of MTZ Release and Its Kinetics

The percentage of drug release from the MTZ@In situ gel was determined, and the percent drug release versus time is shown in Figure 6D. The results show that drug release from the MTZ@In situ gel system occurred in two phases: an initial burst release during solidification, triggered by the rapid exchange of NMP with the surrounding aqueous environment, followed by a phase involving both diffusion and erosion processes [31,32,33], which led to a sustained release pattern of the drug. Approximately 25% and 50% of the drug were released within approximately 3.5 ± 0.02 h and 8.4 ± 0.05 h, respectively, compared to the in-situ gel formulation (release times of T25: 8.99 ± 0.24 h and T50: 21.68 ± 0.58 h, respectively) (Figure 6D). The sustained release pattern in MTZ@In situ gel could be due to the network-like system during solidification, which slowed down the drug release.

The percentage drug release and time data were fitted into different release kinetics models like zero-order, first-order, Higuchi, Korsmeyer–Peppas, and Hixson–Crowell. The DD solver Excel add-in program data are presented in Table 3, suggesting that the drug follows a multiple-release mechanism based on its values. The first-order and Hixson–Crowell models provide the best-fit R^2^ values (R^2^; 0.88 ± 0.03 and 0.90 ± 0.03, respectively). Moreover, drug release follows a non-Fickian transport mechanism (0.45 < n < 0.89).

### 3.10. PK Profile of MTZ and MTZ@In Situ gel

**Figure 7:**
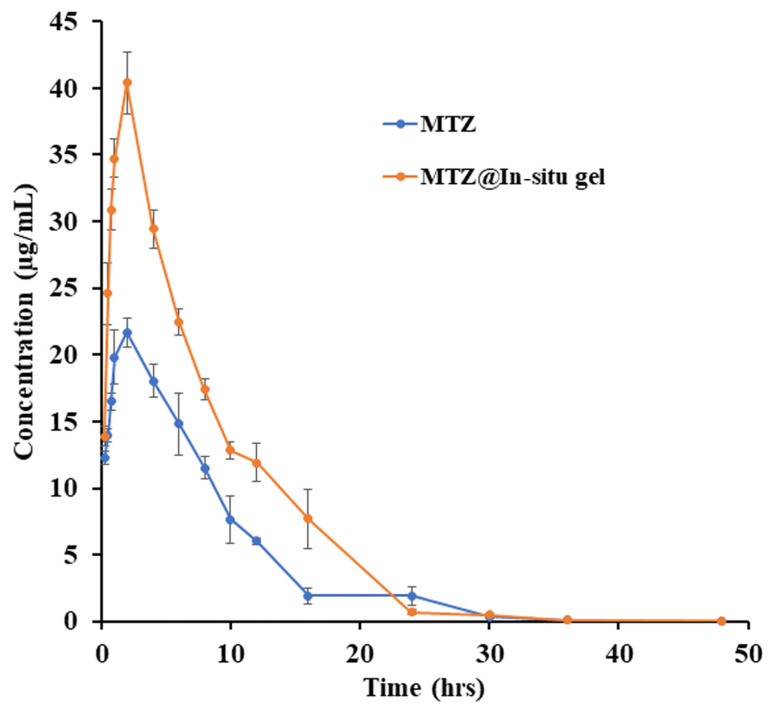
Comparative PK profiles after a single administration of MTZ and the MTZ@In situ gel at 40 mg/rat (n = 4). Data are represented as the mean ± SD.

A PK study was performed in adult rats. MTZ and the MTZ@In situ gel were administered through the s.c. route (drug amount of 40 mg/rat in both groups). The plasma PK parameters are shown in Table 4 and Figure 7. The maximum concentration (C_max_) of MTZ and the MTZ@In situ gel were 22.99 ± 0.87 and 40.40 ± 2.33 ng/mL, with T_max_ of 1.75 ± 0.25 and 2.00 ± 0.00 h, respectively. The half-life of MTZ and the MTZ@In situ gel were 4.39 ± 0.26 and 7.34 ± 1.37 h, respectively. The area under the curve (AUC)_0–last_ of the MTZ@In situ gel was (355.71 ± 22.10 h ng/mL), showing a 73% increase compared to MTZ (205.84 ± 7.05 h ng/mL). The clearance (CL) of the MTZ@In situ gel was 0.45 ± 0.03 L/h/kg, representing a 42.3% decrease compared to MTZ (0.78 ± 0.03 L/h/kg). The relative bioavailability (%F) of the MTZ@In situ gel was 73% higher than that of the free MTZ (Table 4 and Figure 7).

### 3.11. Effect of MTZ@In Situ Gel Formulation on Bone Regeneration at the Osteotomy Site

**Figure 8:**
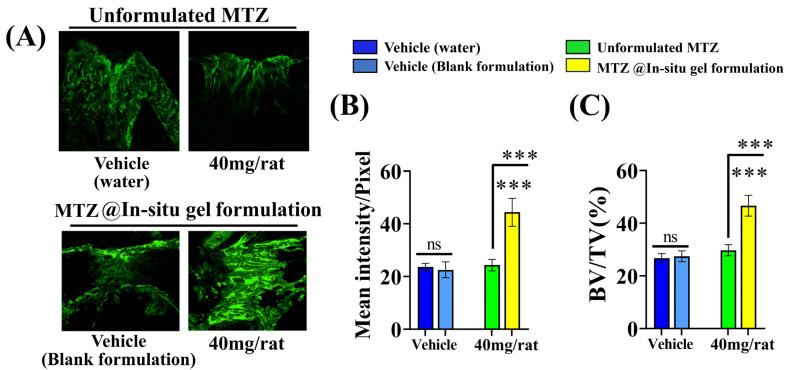
MTZ@In situ gel formulation improved MTZ dose efficacy. (**A**) Representative image shows calcein-labeled image of the fracture site after 12 days of MTZ treatment (MTZ@In situ gel of MTZ and unformulated MTZ) at a dose of 40 mg/rat. (**B**) Quantification data of calcein-labeled newly formed bone at the fracture site after the treatment with the MTZ@In situ gel and unformulated MTZ. (**C**) Callus volume at the fracture site was quantified by µCT after 12 days of the indicated treatments. All values are expressed as the mean ± SEM (n = 6 rats/group). ns, *p* > 0.05 and *** *p* < 0.001 compared to the vehicle (water or MTZ vehicle) or as indicated.

Administering both the unformulated MTZ and MTZ@In situ gel formulation to rats with femur osteotomy at a dose of 40 mg/rat via the s.c. route every three days (totaling four doses over 12 days of the study) showed a remarkable increase (82.26%) in calcein deposition at the osteotomy site compared to the unformulated MTZ. The µCT-based assessment of the BV/TV% at the callus also showed an increase (57.06%) with the MTZ@In situ gel formulation compared to the unformulated MTZ (Figure 8A–C). Considering the total drug exposure of 235 mg/kg administered daily over 12 doses being 564 mg/rat in the initial study (Figure 3), versus every third day for the formulation study (totaling four doses), the potency of the MTZ@In situ gel formulation (total drug exposure of 160 mg/rat) was 3.5-fold higher (Figure 8A–C).

## 4. Discussion

MTZ is widely used in open fractures of extremities to prevent the development of fracture-related infections [34]. Infection after fracture fixation leads to challenging complications, such as non-unions, loss of movement, and, under extreme circumstances, amputation [35]. In our current investigation, the screening of six nitroimidazole drugs revealed MTZ’s osteogenic effect. However, it did not modify OVX-induced bone resorption, resembling the action of osteogenic drugs like teriparatide and abaloparatide. The osteogenic action of MTZ, without affecting bone resorption, has an advantage during fracture healing because it enables the continuation of normal bone remodeling processes. As a result, the initial deposition of woven bone at the fracture site that must undergo resorption by osteoclasts can progress unhindered into mature lamellar bone formation, ultimately restoring bone strength and integrity during fracture healing. Common infections affecting bones, which lead to bone loss, can significantly impair fracture healing.

Osteomyelitis, septic arthritis, and periodontitis are, respectively, common infections of bones, joints, and gums and cause bone loss [36]. These infections can occur spontaneously as well as through surgical procedures. Inflammation is the direct sequela of infections, which could be either systemic or local (bone, joint, or gum). Regardless of the site of inflammation, the outcomes include the activation of osteoclasts and the inhibition of osteoblast function, thus resulting in bone loss [37]. Antibiotics are thought to facilitate bone healing by suppressing infection, although some of these, including gentamicin, cephalosporin, and tetracyclin, are known to inhibit osteoblast function [11,12,38]. An antibiotic with bone-anabolic effects offers significant advantages for orthopedic and dental operative procedures. Firstly, the dual action of infection prevention and bone regeneration reduces the need for additional medications. Additionally, the dual action enhances overall patient outcomes by facilitating the faster healing of bone tissue, reducing recovery time, and lowering the risk of complications such as non-unions or revision surgeries. Imidazole group compounds have been studied for their effects on bone cells, with a primary focus on osteoclasts (discussed in the next paragraph). However, in fracture healing, enhancing osteoblast function is critical, making the discovery of imidazole compounds with osteogenic potential promising.

Imidazole compounds have been reported to have both anti-resorptive and pro-resorptive effects, although there is no report of their osteogenic effect. Regarding the anti-resorptive effect, 2-([1,1′-biphenyl]-4-yl)-1-(2-(piperidine-1-yl)ethyl)-1H-benzo[d]imidazo[1,2-a]imidazole protected mice against inflammation-induced bone loss by suppressing osteoclastogenesis [39]. In addition, 1-methyl-imidazole and benzimidazole inhibited ex vivo bone resorption by suppressing thromboxane-A2 formation [40], and 5-chloro-2-ethyl-4-nitro-1-propyl-1H-imidazole inhibited RANKL-induced osteoclastogenesis in murine osteoclast precursor cells, RAW264.7 [41]. On the other hand, azathioprine, a purine-based imidazole derivative used as an adjunct immunosuppressor in renal transplantation, increased the osteoclast surface, an indicator of active bone resorption [42], and also enhanced bone loss in an IBD model of mouse [43]. Unlike the aforementioned imidazole compounds that inhibit osteoclast formation, we found that MTZ stimulated osteoblast function. Ethanidazole also showed an in vitro osteogenic effect, although it was much less potent than that of MTZ. The osteogenic effect observed in MTZ and ethanidazole is likely attributed to the presence of a nitroimidazole ring. The presence of an ethyl group attached to the nitrogen atom of the imidazole ring, as seen in ethanidazole, appears to have attenuated the osteogenic effect compared to MTZ, which lacks this group. Secnidazole, ronidazole, tinidazole, and ornidazole lack the nitroimidazole ring and do not exhibit osteogenic effects, highlighting the essential role of the nitroimidazole ring in promoting osteogenesis. From these findings, we propose synthesizing novel compounds with nitro group-containing imidazole compounds to further enhance potency in promoting osteogenesis and combating infections for critical size defects that often lead to non-unions.

The activation of the Wnt pathway has an osteogenic effect [44] that promotes fracture healing and new bone formation in OVX rats treated with MTZ. The osteogenic effect of Wnt signaling has been established by the demonstration of individuals with LRP-5/6 mutations having skeletal phenotypes. Gain-of-function mutations in LRP5 result in a high bone mass phenotype in mice [45], while loss-of-function mutations in LRP5 and LRP6 lead to decreased bone mass [46]. The activation of the Wnt pathway triggers downstream events, including the increased expression of critical osteogenic markers, such as Runx2 and BMP-2, in MSCs [47]. Runx2 is a master transcription factor known to regulate osteoblast differentiation and bone formation, while BMP-2 is a key growth factor that promotes bone regeneration [48]. The upregulation of these two genes in BMSCs by MTZ suggests the stimulatory effect of the drug on the commitment of BMSCs to the osteogenic lineage and augments their capacity to produce new bone tissue. Consequently, the activation of the Wnt pathway and the subsequent upregulation of Runx2 and BMP-2 levels contribute significantly to the acceleration of fracture healing and the overall bone anabolic effects observed in OVX rats treated with MTZ. In contrast to the activation of Wnt/β-catenin by MTZ, pyridinylimidazoles downregulate melanogenesis via the suppression of Wnt/β-catenin signaling [49]. Thus, it appears that, within the imidazole group of compounds, the presence of the nitroso group, as found in MTZ, activates the Wnt pathway. In contrast, fusing a pyridine ring with the imidazole ring inhibits the Wnt pathway. This result suggests that the nitroso group within the imidazole compounds can promote the Wnt pathway. It would be interesting to study whether the suppression of Wnt/β-catenin signaling by pyridinylimidazoles leads to the inhibition of bone formation in vivo.

While MTZ showed Wnt signaling activation in osteoblasts in vitro and promoted fracture healing, the in vivo dose used was equivalent to human dosing. Given its usual one-week administration, its longer use for prophylaxis against non-union in fractures requires addressing its potential toxicity by reducing the effective dose through methods such as an in situ gel formulation. This type of formulation offers several advantages for drug delivery, including controlled release, improved bioavailability, reduced dosing frequency, and enhanced patient compliance [50]. These formulations have been applied in various therapeutic areas, including localized treatment of different diseases. The gel depot provides localized drug delivery, minimizing systemic exposure and potential side effects [51]. Additionally, it can enhance the bioavailability of poorly soluble drugs by maintaining them in a solubilized state within the gel matrix. The MTZ@In situ gel formulation is a type of drug delivery system designed to undergo a phase transition from a solution to a gel upon administration at the target site. The particle size and PDI were 164 ± 13.68 and 0.29 ± 0.04, respectively. The drug content was 90 ± 0.254%, which may be attributed to incorporation of MTZ in the matrix gel. We observed that MTZ was released up to 80% within 24 h. The release exponent value (n > 0.5) indicates a non-Fickian diffusion mechanism, i.e., diffusion followed by erosion [52,53]. This sustained release profile helps to maintain therapeutic drug levels in the body for longer periods. The sustained release characteristic of the MTZ@In situ gel formulation reduced the drug administration frequency, potentially leading to improved patient compliance and decreased high dose-related toxicity.

The generation of anionic intermediates primarily contributes to MTZ-induced toxicity, with neurological and genotoxic effects being the most notable. Reducing the drug dose is a key strategy for diminishing toxicity. In our formulation, NMP acts as the solvent of MTZ, and biocompatible polymer (e.g., mPEG-PDLLA) forms the depot. When injected, the solution undergoes a temperature-sensitive transformation into a gel at body temperature, enabling the controlled and sustained release of MTZ. In situ gel-forming systems, of which mPEG–PDLLA is an example, offer a promising alternative to long-acting injectables like disperse systems and microspheres due to their biocompatibility, stability, ease of administration, and scalability. The mPEG–PDLLA-based in situ gel system has been effectively used to deliver tinidazole for the experimental treatment of periodontal inflammation induced by P. gingivalis in rabbits [54]. Moreover, mPEG–PDLLA is considered safe and has been approved by the U.S. FDA for clinical use [55]. The favorable drug release kinetics and excellent in vivo characteristics make MTZ@In situ gel suitable for MTZ delivery. The PK study revealed that MTZ bioavailability parameters were significantly enhanced in the MTZ@In situ gel compared to the unformulated MTZ, including increases in Cmax (75%) and AUC (72%) and a decrease in Cl (43%), which collectively resulted in a 73% increase in relative bioavailability, culminating in a 3.5-fold increase in the fracture healing efficacy. These data underscore the potential of the MTZ@In situ gel formulation as a promising therapeutic approach for improving bone regeneration and fracture outcomes without the safety concerns associated with this drug.

However, our study has caveats. We did not evaluate the efficacy of the MTZ@In situ gel formulation in a critical-sized defect model (representing compound fractures in humans) or in osteopenic rats. Also, we did not assess the quality of the regenerated bone in response to MTZ and the MTZ@In situ gel formulation. Finally, while MTZ is typically prescribed for about a week, a longer treatment may be necessary to prevent infection and non-union at compound fracture sites. Therefore, assessing the long-term safety and tolerability of MTZ is imperative. Although our formulation reduces the MTZ dose by 3.5-fold, its potential long-term toxicity remains a concern and warrants further investigation in future studies.

## 5. Conclusions

Our study revealed the osteogenic potential of MTZ, attributed to its activation of the canonical Wnt pathway. Moreover, we identified a promising avenue for repurposing MTZ to facilitate bone regeneration in patients with fractures through an injectable MTZ@In situ gel formulation, which displays superior PK profiles and fracture-healing efficacy over the unformulated MTZ, potentially mitigating the toxicity risks associated with this drug. Based on our findings, the MTZ@In situ gel formulation appears to be a promising prophylactic approach for lowering infection risk in comminuted fractures. Implementing this method may significantly minimize the number of postoperative infections, reducing the need for revision procedures. This has important implications for improving patient outcomes, cutting healthcare costs, and increasing the overall efficacy of fracture management techniques.

## Figures and Tables

**Table 1:** Primer sequence of the various genes used for qPCR.

Gene Name	Primer Sequence
Species: Rat	
Runx2	F-CCACAGAGCTATTAAAGTGACAGTG
	R-AACAAACTAGGTTTAGAGTCATCAAGC
BMP2	F-CGGACTGCGGTCTCCTAA
	R-GGGGAAGCAGCAACACTAGA
TRAP	F-CAGGGACGGGAGAGATTGG
	R-GCTGTACAGTGAGCCAGGA
GAPDH	F-TGGGAAGCTGGTCATCAAC
	R-GCATCACCCCATTTGATGTT
Species: Mouse	
Runx2	F-TTGACCTTTGTCCCAATGC
	R-AGGTTGGAGGCACACATAGG
BMP2	F-AGATCTGTACCGCAGGCACT
	R-GTTCCTCCACGGCTTCTTC
GAPDH	F-AGGGATGCTGCCCTTACC
	R-TCTACGGGACGAGGAAACAC

F: forward primer; R: reverse primer.

**Table 2:** ALP screening of imidazole drugs.

Compounds	Activity in ALP Assay	Structure
Etanidazole	Positive (EC50: 400 nM)	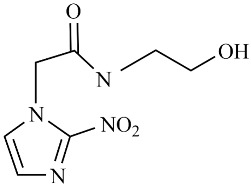
Secnidazole	Negative (EC50: >10 μM)	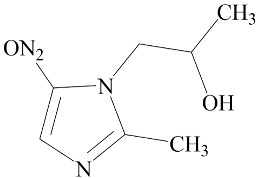
Ronidazole	Negative (EC50: >10 μM)	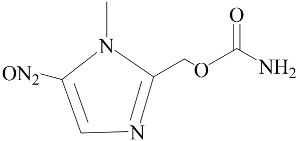
Metronidazole	Positive (EC50: 1.2 nM)	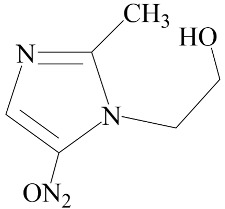
Tinidazole	Negative (EC50: >10 μM)	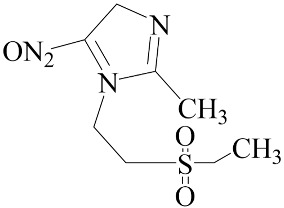
Ornidazole	Negative (EC50: >10 μM)	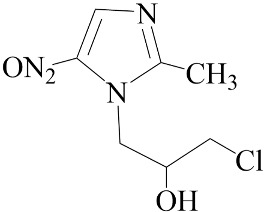

**Table 3:** Release kinetic parameters for the in situ gel formulation (MTZ@In situ gel).

Release Kinetics	Parameters	MTZ in PBS	MTZ@In Situ Gel in PBS
Zero-order	K_0_	4.77 ± 0.05	2.43 ± 0.04
	R^2^	0.76 ± 0.01	0.83 ± 0.04
First-order	K_1_	0.08 ± 0.00	0.03 ± 0.00
	R^2^	0.84 ± 0.00	0.90 ± 0.03
Higuchi	k_H_	16.34 ± 0.12	8.27 ± 0.18
	R^2^	0.71 ± 0.00	0.76 ± 0.01
Korsmeyer–Peppas	kKP	8.64 ± 0.11	4.15 ± 0.46
	n	0.78 ± 0.01	0.80 ± 0.04
	R^2^	0.81 ± 0.01	0.87 ± 0.03
Hixson–Crowell	kHC	0.02 ± 0.00	0.01 ± 0.00
	R^2^	0.86 ± 0.01	0.88 ± 0.03

K_0_, K_1_, K_H_, kKP, and kHC are the release rate constants for the zero-order, first-order, Higuchi, Korsmeyer–Peppas, and Hixson–Crowell models, and n is the release exponent.

**Table 4:** PK profiles of MTZ and MTZ@In situ gel (40 mg/rat) administered by the s.c. route to rats (n = 4, mean ± SEM).

PK Parameter	MTZ	MTZ@In Situ Gel
t_1/2_ (h)	4.39 ± 0.26	7.34 ± 1.37
T_max_ (h)	1.75 ± 0.25	2.00 ± 0.00
C_max_ (µg/mL)	22.99 ± 0.87	40.40 ± 2.33 ***
AUC _0–∞_ (h µg/mL)	205.84 ± 7.05	355.71 ± 22.10 ***
Vd (L/kg)	4.91 ± 0.15	4.95 ± 1.11
Cl (L/h/kg)	0.78 ± 0.03	0.45 ± 0.03 ***
MRT (h)	7.63 ± 0.30	7.44 ± 0.27
Relative bioavailability (%F)	100	172.62 ± 7.29

*** *p* < 0.001 compared to the MTZ group.

## Data Availability

The raw data supporting the conclusions of this article will be made available by the authors upon request.
